# Assessing HIV transmission knowledge in psychiatric patients in Johannesburg, South Africa

**DOI:** 10.4102/sajpsychiatry.v29i0.2040

**Published:** 2023-06-30

**Authors:** Hangwani J. Matodzi, Karishma Lowton, Prinesh Miseer

**Affiliations:** 1 Department of Psychiatry, Faculty of Health Sciences, University of the Witwatersrand, Johannesburg, South Africa

**Keywords:** HIV knowledge, KQ-18, psychiatric patients, South Africa, HIV transmission, HIV prevention, mental illness

## Abstract

**Background:**

The bidirectional relationship between human immunodeficiency virus (HIV) and psychiatric illnesses is well documented. Misinformation about HIV transmission and prevention is associated with high rates of HIV-related risky behaviours, and therefore, HIV infection risk.

**Aim:**

To assess basic HIV transmission knowledge in psychiatric patients.

**Setting:**

Outpatient psychiatric clinic at Tara Psychiatric Hospital, Johannesburg, South Africa.

**Methods:**

A cross-sectional, quantitative study was conducted employing a self-administered HIV knowledge questionnaire, the 18- item HIV knowledge questionnaire (HIV-KQ18). Consent, demographic, and clinical profile information were obtained from participants meeting the selection criteria.

**Results:**

This study indicated a mean knowledge score of 12.6 (69.7%) out of 18, and therefore good knowledge. The highest HIV-KQ18 mean scores were found in patients with personality disorders (78.9%), anxiety disorders (75.6%) and bipolar and related disorders (71.1%). Participants with schizophrenia, depressive disorders and substance use disorders had scores ranging between 66.1% and 69.4%. Statistically significant differences in knowledge were evident based on age, marital status, level of education and employment status. Interestingly, participants who used substances had higher average basic HIV transmission knowledge scores compared to those who did not use substances.

**Conclusion:**

Good overall HIV transmission knowledge was found in this population, albeit lower than in the general population. Statistically, correlates were found between psychiatric diagnosis, substance use, age, marital status, level of education, and employment status and basic level of HIV knowledge.

**Contribution:**

HIV knowledge remains lower in psychiatric patients than in the general population, with correlates between demographic and clinical factors, calling for psychoeducation efforts to take all these into consideration.

## Introduction

Human immunodeficiency virus (HIV) prevalence among adults with serious mental illness in Africa, where the HIV disease burden is higher compared to other continents, is said to range between 11% and 27%.^1,2,3^ People at risk of, or who have HIV are often at risk of mental illness and often encounter other significant biopsychosocial challenges that impact negatively on their access and adherence to HIV prevention and treatment modalities.^4^

Mental health disorders have been shown to critically contribute to HIV infection, increasing the risk of transmission by 4–10-fold.^5,6^ These findings have been replicated internationally with factors such as impaired perception to reality, emotional instability and impulsivity contributing to such behaviours; however, poor knowledge about HIV infection is also an important factor.^7^

Recent literature has revealed that psychiatric patients, specifically those who have been diagnosed with schizophrenia, may be at high risk for HIV infection.^8^ However, substance use disorders, particularly alcohol, are the most common mental disorders amongst HIV infected people.^9^ Some mental health conditions, such as depression, may make HIV management complicated, leading to poor HIV treatment adherence and compromised secondary prevention efforts.^10^

One is considered to have HIV knowledge if they have the correct information pertaining modes of transmission, high risk behaviour, and prevention and available management strategies.^11^ Lower levels of HIV knowledge have been found among mentally ill patients compared to non-psychiatric patients.^12,13,14^ Additionally, poor knowledge or misinformation, was linked to high rates of reporting of HIV-related risky behaviours.^15^ An individual’s knowledge of HIV transmission and accurate risk assessment are important in adopting safer sexual practices.^16^ Furthermore, it is important to educate individuals about myths associated with HIV as partial knowledge can increase, and maintain, the risk of infection.^17^

Knowledge questionnaires have been used to assess HIV knowledge in mentally ill patients. Grassi et al.^8^ in their study performed in Ferrara, in north-eastern Italy, used the acquired immunodeficiency syndrome (AIDIS) Knowledge Test (AIDS-KT) and revealed fair HIV knowledge in 51% of a population with mental illness as compared to 91% in the control group. The AIDS-KT instrument used by Grassi et al. is an 11-item true-false self-reporting questionnaire that is designed to assess accurate HIV transmission and prevention knowledge among psychiatric patients.

Another study conducted in India, Chandra et al.^18^ assessed knowledge regarding HIV transmission and prevention in patients diagnosed with severe mental illness using the 18-item HIV knowledge questionnaire (HIV-KQ18). Research indicated that the HIV-KQ18 has been used and validated in psychiatric patients. It has been demonstrated that this instrument is internally consistent, and stable. It has also been found to be sensitive to change resulting from intervention and could be used in populations of low education levels.^11^ In utilising this scale, a score of less than 50% is said to be an indication of significant lack of knowledge, extreme misconceptions about HIV transmission, and in a case where a participant may be guessing, bad luck.^11^

In their study, Chandra et al.^18^ assessed baseline knowledge regarding HIV and AIDS and knowledge retention after 5 days post-HIV education. Their study revealed poor baseline knowledge, after which there was improvement for 5 days following a HIV educational programme. Their findings indicated that HIV-focused training programmes, albeit short, may lead to improvement in HIV knowledge in Indian psychiatric patients.

We opted to use the HIV-KQ18 in this study to assess the level of basic HIV and AIDS transmission knowledge in mental health care users. This may assist in structuring targeted interventions that are geared for the mentally ill, which can be integrated into existing mental health psychoeducation programmes.

## Research methods and design

### Study setting and participants

This study was a cross-sectional, quantitative study employing the use of a self-administered basic HIV knowledge questionnaire, the HIV-KQ18.

This study was carried out at the outpatient department (OPD) at the Tara H Moross Psychiatric Hospital (Tara), a specialist psychiatric hospital situated in Johannesburg, South Africa. The sample population consisted of consenting psychiatric patients who met the inclusion criteria, at the OPD.

The inclusion criteria were mental health care users aged 18 years and above of all genders who had been diagnosed with a psychiatric condition and were psychiatrically stable at their last review. Participants had to have a basic understanding of the English language and must have expressed willingness to participate in the study.

The exclusion criteria were mental health care users under the age of 18 years and mental health care users who were unable or unwilling to provide consent to participate in the study, acutely unwell mental health care users and patients without basic English understanding.

### Sample size and sampling strategy

Tara services an estimated 4200 mental health care users each year, both inpatients and outpatients. The outpatient to inpatient population ratio was 6:1 with a recorded 2337 mental health care service users seen at the OPD clinics over a 6 month period from July 2019 to December 2019.

Although Tara Hospital’s services include specialised mental health care for users with child, adolescent, eating and neuropsychiatric disorders, these OPD units are separate from the one where the study was conducted, allowing the focus to be placed on general psychiatric patients. Patients who follow up at this OPD vary in terms of psychiatric profiles and follow-ups are arranged in a similar manner compared to other tertiary level psychiatric hospital OPDs.

The minimum sample size for this study was calculated assuming a population size of 2337 over a 6 month period. This figure related to the number of patients seen and not the number of follow-up visits. With a 50% expected frequency for the outcome, a 5% significance level and a single cluster, a minimum sample size of 330 was reached assuming a 95 % confidence level. A 10% contingency for a possible non-response rate was included, amounting to 33 additional participants, giving a final minimum sample size of 363. The actual sample size was 371.

Purposive quota sampling was used. Participation was specifically for the purposes of this research, and sampling ceased once the required quota of participants was reached. Data collection was conducted over a period of 3 months, from January 2022 to March 2022.

### Research instrument

A three sectioned data collection instrument was used. The first section requested demographic information including age, gender, marital status, race, employment status and level of education. The second section included the primary *Diagnostic and Statistical Manual of Mental Disorders, Fifth Edition* (DSM-V) diagnosis, drug use, whether the participant had HIV counselling, and known HIV status. The third section was the HIV-KQ18.

The HIV-KQ18 is an 18-item HIV knowledge questionnaire that has been used and validated in psychiatric patients.^11^ In employing this scale, scores below 50% suggest significantly poor knowledge, total misunderstanding or false beliefs about HIV transmission.^8^

Permission to utilise the HIV-KQ18 was obtained from the lead creator of the tool, Professor Michael P. Carey (The Alpert Medical School of Brown University).

### Data collection

The Tara OPD staff, the doctors, nurses and administration clerks were all briefed about the study and data collection process. Posters and pamphlets with information about the study were placed in the OPD waiting area to recruit and ensure ease of access of information regarding the study.

Once ethical clearance and permission to conduct the study were obtained, data collection began. Participants were first required to provide written consent due to the perceived sensitivity of the topic, especially considering that questions on the HIV status of the individuals were asked. Participants then answered the demographic and clinical questions and completed the HIV-KQ18. Completed forms were placed in clearly marked allocated boxes at the reception area.

### Data analysis

Data were captured by copying all responses on the questionnaires directly on to an Excel spreadsheet. For the descriptive analysis, categorical data were reported as frequencies and percentages while continuous data were reported as means and standard deviations.

To compare the means of two groups, an independent sample *t*-test was computed, while to compare the means of three or more groups a one-way analysis of variance (ANOVA) was computed. All statistical analyses were run using the 2018 JASP statistical package.

### Ethical considerations

The Wits Human Research Ethics Committee provided ethical clearance and approval for this study, certificate reference number R14/49, protocol number M210446. Permission to conduct the study was granted by the Chief Executive Officer of the Tara H Moross Psychiatric Hospital.

In conducting this study, care was taken to ensure voluntary participation with written informed consent, confidentiality, anonymity, and voluntary withdrawal at any point during the data collection. A distress protocol was available, ensuring availability of the principal investigator, as a qualified counsellor, to provide counselling and support.

## Results

A total of 371 psychiatric patients participated in this study. Information about their demographic variables (age, gender, marital status, race, employment status and level of education) was obtained, as well as clinical variables (primary DSM-V diagnosis, drug use, exposure to HIV counselling, and known HIV status). All 371 participants completed the HIV-KQ18. The mean number of answers correct out of a possible 18 on the HIV-KQ18 was 12.6 equivalent to 69.7%, with 14 % (*n* = 52) of participants demonstrating poor basic HIV transmission knowledge, with a score < 50%. More than 80% of the participants had adequate basic HIV transmission related knowledge.

The demographic profile of the sample together with HIV-KQ18 scores may be seen in Table 1.

**TABLE 1:** Demographic profile.

Variables	*n*	%	Mean HIV-KQ18 score	Mean HIV-KQ18 score (s.d.)	HIV-KQ18 score as %	*p* *
**Age group**						
18 to 19	4	1.1	12.8	1.5	71.1	< 0.001
20 to 29	61	16.4	12.0	3.5	66.7	-
30 to 39	85	22.9	13.7	3.2	76.1	-
40 to 49	82	22.1	12.9	4.0	71.7	-
50 to 59	83	22.4	13.1	3.9	72.8	-
60 to 69	39	10.5	11.1	4.6	61.7	-
70 to 81	16	4.3	9.0	4.6	50.0	-
**Race**						
Black people	89	24.0	13.2	3.5	73.3	0.190
Coloured people	17	4.6	13.4	4.5	74.4	-
Indian people	18	4.9	11.2	5.7	62.2	-
White people	240	64.7	12.5	3.8	69.4	-
Other	7	1.9	11.0	6.2	61.1	-
**Gender**						
Female	223	60.1	12.5	4.1	69.4	0.847
Male	146	39.4	12.7	3.7	70.6	-
Other	2	0.5	13.5	0.7	75.0	-
**Marital status**						
Cohabiting	17	4.6	14.1	3.1	78.3	0.025
Divorced	55	14.8	13.2	3.4	73.3	-
Married	67	18.1	12.6	4.4	70.0	-
Separated	8	2.2	13.8	5.5	76.7	-
Single	212	57.1	12.5	3.7	69.4	-
Widowed	12	3.2	9.3	5.9	51.7	-
**Level of education**						
Less than matric	67	18.1	11.0	4.3	61.1	< 0.001
Matric	123	33.2	11.6	4.3	64.4	-
Post-Matric Diploma	85	22.9	13.6	2.9	75.6	-
University	96	25.9	14.2	3.1	78.9	-
**Employment status**						
Employed	129	34.8	13.5	3.4	75.0	0.001
Unemployed	242	65.2	12.2	4.1	67.8	-

* *p*-value < 0.05 denotes statistical significance.

HIV, human immunodeficiency virus; s.d., standard deviation; HIV-KQ18, 18- item HIV knowledge questionnaire.

### Age

The mean age for the entire sample was 44.2 years. From Table 1, most participants were 30 to 39 years old (*n* = 85), followed by 50 to 59 years old (*n* = 83) and 40 to 49 years old (*n* = 82). When the age groups were compared on their HIV knowledge via the KQ18 using an ANOVA, the results were statistically significant (*F* [3, 364] = 5313; *p* < 0.001). This indicated that the participants aged 70 and over (*n* = 16) had a far lower knowledge of HIV compared to all the younger age groups, indicated by the mean which was found to be much lower than the younger groups.

### Gender

From Table 1, the sample comprised 223 females (60.1%) and 145 males (39.4%), while two (0.5%) considered their gender to be ‘other’. When the genders were compared on their HIV knowledge via the KQ18, the results were not statistically significant (*p* = 0.847) indicating no gender differences in HIV knowledge.

### Race

From Table 1, white participants (*n* = 240) and black participants (*n* = 89) made up most of the sample, followed by Indian (*n* = 18), Coloured (*n* = 17) and ‘other’ (*n* = 7). A comparison of HIV knowledge using the KQ18 across the race groups yielded non-significant differences (*p* = 0.190).

### Marital status

From Table 1, most of the participants were single (*n* = 212), married (*n* = 67), or divorced (*n* = 55). When the marital statuses were compared on their HIV knowledge via the KQ18 using an ANOVA, the results were statistically significant (*F* [5, 365] = 2608; *p* < 0.025, indicating the lowest average score (51.7%) in widowed participants. Co-habiting participants scored the highest average score of 78.3%.

### Level of education

From Table 1, most of the participants had a matric (*n* = 123), with many having a diploma (*n* = 85) or a degree (*n* = 95), and the lowest number not having a matric (*n* = 67). When the educational levels were compared on their HIV knowledge via the KQ18 using an ANOVA, the results were statistically significant (*F* [3, 367] = 15 555; *p* < 0.001). This indicated that the participants differed in their HIV knowledge according to education level, with those without matric having the lowest mean of 11.0 (standard deviation [s.d.] = 4.3) and those with a matric only having slightly more knowledge with a mean of 11.6 (s.d. = 4.3). Those with a diploma had a better mean of 13.6 (s.d. = 2.9) while those with a degree had the highest mean of 14.2 (s.d. = 3.1).

### Employment status

From Table 1, approximately two-thirds (*n* = 242) of the sample were unemployed and roughly one-third were employed (*n* = 129). An independent *t*-test to compare the two groups indicated statistical significance (*t* [369] = 3215; *p* = 0.001). Those with employment had a higher level of knowledge than those who were unemployed.

### Clinical profile

The clinical profile of the sample together with HIV-KQ18 scores may be seen in Table 2.

**TABLE 2:** Clinical profile.

Variable	*n*	%	Mean HIV-KQ18 score	s.d.	HIV-KQ18 Score as %	* *p*
**Primary DSM-V diagnosis**						
Anxiety disorders	5	1.3	13.6	3.8	75.6	0.007
Bipolar and related disorders	129	34.8	12.8	3.8	71.1	-
Depressive disorders	83	22.4	12.2	4.1	67.8	-
Personality disorders	44	11.9	14.2	2.0	78.9	-
Schizophrenia spectrum and other psychotic disorders	78	21.0	12.5	4.3	69.4	-
Substance related and addictive disorders	7	1.9	11.9	3.4	66.1	-
Diagnosis unknown	5	1.3	7.6	3.7	42.2	-
Other	20	5.4	11.5	4.7	63.9	-
**Drug use**						
No	241	65.0	12.0	4.4	66.7	< 0.001
Yes	130	35.0	13.8	2.6	76.7	-
**HIV counselling**						
No	310	83.6	12.3	4.1	68.3	< 0.001
Yes	61	16.4	14.2	2.9	78.9	-
**HIV status**						
Negative	58	15.6	14.3	2.8	79.4	0.001
Positive	6	1.6	13.5	3.4	75.0	-
Unknown	307	82.7	12.3	4.1	68.3	-

* *p*-value of < 0.05 denotes statistical significance.

HIV, human immunodeficiency virus; s.d., standard deviation; HIV-KQ18, 18- item HIV knowledge questionnaire; DSM-V, diagnostic and statistical manual of mental disorders, fifth edition.

### Primary DSM-V diagnosis

From Table 2, the most frequent diagnoses were bipolar and related disorders (*n* = 129), depressive disorders (*n* = 83), and schizophrenia spectrum and other psychotic disorders (*n* = 78). An ANOVA to compare all diagnostic groups on their HIV knowledge using the KQ18 reached statistical significance (*F* [7, 363] = 2841; *p* = 0.007). The difference could be attributed to the relatively low HIV-KQ18 mean score of 7.6 (s.d. = 3.7) for the very small group (*n* = 5) of those with unknown diagnosis. The highest KQ18 means were 14.2 (s.d. = 2.0) for those with personality disorders (*n* = 44) and 13.6 (s.d. = 3.8) for those with anxiety disorders (*n* = 5), again a very small sample.

### Drug use

From Table 2, approximately two-thirds of participants did not use drugs (*n* = 241) while approximately one-third did use drugs (*n* = 130). An independent *t*-test to compare the two groups indicated statistical significance (*t* [369] = −4.447; *p* < 0.001). Those who used drugs had a higher HIV knowledge.

### HIV counselling

From Table 2, most of the participants had not received HIV counselling (*n* = 210) and 16.4% (*n* = 61) had. An independent *t*-test to compare the two groups indicated statistical significance (*t* [369] = −3.482; *p* < 0.001). Those who had had HIV counselling had a higher HIV knowledge than those who had not.

### HIV status

From Table 2, the HIV status was unknown in most cases (*n* = 307). There were six HIV-positive and 58 HIV-negative participants. An ANOVA to compare these groups on their HIV knowledge using the KQ18 reached statistical significance (*F* [2, 368] = 6860; *p* = 0.001). Those who were HIV-negative displayed better HIV knowledge.

## Discussion

In a study conducted in Soweto, Johannesburg, the researchers used a 63-item questionnaire. The study revealed that approximately 50% of the psychiatric patients assessed had good knowledge of HIV and AIDS.^19^ A number of studies have also been conducted in developed countries, and these have indicated poorer HIV and AIDS knowledge in psychiatric patients than the general population.^12,14,20^ However, some studies have reported higher knowledge scores to HIV questionnaires, ranging from 63% to 80%,^13,21,22^ comparable to scores seen in the general population.^23^

This study demonstrated that 14% (*n* = 52) of participants had poor basic HIV transmission knowledge, scoring < 50%. More than 80% of the participants had adequate HIV-related knowledge, with the mean score at 69.7%. Such a level of HIV knowledge is not the lowest reported among psychiatric patients; however, with HIV and AIDS poor knowledge associated with high rates of reporting HIV-related risky behaviours,^15^ it remains evident that efforts at improving basic HIV and AIDS transmission knowledge in the mentally ill are warranted.

### Gender and race

In their study to determine knowledge, attitudes and personal beliefs regarding HIV and AIDS in psychiatric patients at clinics in Soweto, Johannesburg. Jonsson et al.^19^ found that female patients had relatively better knowledge about HIV and AIDS. Whereas Shamu et al.^24^ in their study done in non-psychiatric persons in the Kangala District of Mpumalanga and OR Tambo District in the Eastern Cape, found that women were less knowledgeable compared to men in both districts, however, much lower in OR Tambo district. In comparing genders and race on their HIV knowledge using the KQ-18 did not reveal statistically significant results, (*p* = 0.847) and (0.190), respectively, indicating no gender nor race differences in HIV knowledge. With regards to gender, this finding was consistent with the findings of the Katz study where males and females did not differ in AIDS knowledge.^25^ With the high HIV prevalence in South Africa, and highest amongst black South Africans, there have been active HIV education drives to educate the public about HIV and AIDS, Tuberculosis (TB) and sexually transmitted infections (STIs), mainly targeting the previously disadvantaged populations. This may explain the same knowledge level across races in our study.

### Level of education

Some studies have shown a relationship between higher educational levels and HIV and AIDS knowledge, where persons with higher levels of education showed better HIV and AIDS knowledge.^3,26^ The South African school curriculum offers sexuality and HIV education as part of life orientation as a school subject. However, this has come with challenges ranging from educators’ levels of HIV knowledge,^27^ educators’ attitudes and feelings towards sexual health education, and commonly, reluctance to hold discussions about sexuality.^28^ This may explain the findings in this study, which indicated that the participants differed in their HIV knowledge according to level of education, with those with a university qualification scoring highest, and the lower scores found in participants with less than matric.

However, in a study done at Weskoppies, a South African psychiatric hospital in Pretoria, findings revealed no significant statistical difference in knowledge when participants with a level of education less than high school were compared with those whose level of education was more than high school. In contrast, the participants were inpatients; therefore, the authors noted that this could be explained by the fact that participants had underwent multiple sexual health education sessions during their long stay in the hospital as part of routine care.^29^

### Marital status

A South African study by Shisana et al.^30^ revealed that about 72.72% of married participants perceived themselves to be definitely not at risk of HIV infection compared to unmarried persons.

This low perception of risk may be a major reason for not testing,^31^ and therefore, poor HIV knowledge as testing is associated with counselling and education around HIV transmission and risk. The lack of awareness of their risk may increase their lack of knowledge, and in turn resulting in low condom use, and therefore, associated increased risk of acquiring HIV.^32^

### Employment status

Service users who reported employment had a higher level of knowledge than those who reported unemployment. This could be explained by HIV and AIDS workplace programmes in some workplaces, with HIV policies and programmes including education programmes and provision of voluntary counselling and testing.^33^ Conyers et al.^34^ published a critical review which concluded that there is enough evidence to support a direct relationship between employment and HIV prevention, suggesting that employment might play a role in HIV transmission reduction.

### Drug use

With the global HIV prevalence in people who inject drugs estimated at 17.6%,^35^ some harm reduction strategies have been implemented in some cities, and these include outreach programmes for substance users or ex-users; access to sterile equipment and cleaning materials, and information about safer drug use and safer sex; counselling, support groups.^4^ Despite all these efforts, this study has revealed that service users had lower basic HIV knowledge. This may be explained by several factors, including co-morbid psychiatric disorders, with cognitive deficits, and lack of access to some of these available services offered to substance users or ex-users. Access to these services may also be hindered by time, where attending a mental health clinic takes priority to attending platforms that provide support and psychoeducation on substance use.

### HIV status

This study showed that those who were HIV negative had better HIV knowledge compared to those who were HIV positive or did not know their HIV status. The South African National HIV Counselling and Testing (HCT) Policy Guidelines recommend that all sexually active persons should be encouraged to test at least annually, and this should be promoted as part of the culture of proactive self-care that individuals should adopt.^36^ As part of the testing process, pre-test counselling includes information about HIV acquisition and transmission. This would explain the findings in this study, where mental health care users who knew their HIV status to be negative, with regular HIV counselling and testing, may demonstrate adequate knowledge in HIV transmission, more so as emphasis is made on preventing HIV infection in those who test HIV negative, recommending a repeat test after 3 months to exclude the window period, and thereafter testing annually.

### Study limitations

A number of limitations should be noted for this study. The tool used in this study is a self-reporting questionnaire, and information obtained from these types of tools is dependent on the motivation of the participants to provide truthful and accurate answers, which may lead to non-response bias.

The knowledge questionnaire used in this study was developed and validated in psychiatric patients in the United States of America, however, not specifically validated in psychiatric patients in South Africa. The few studies conducted in South Africa assessed HIV knowledge with the use of different questionnaires, for example, the AIDS-KT. This may limit comparison of this study with available literature.

Despite the high prevalence of HIV in South Africa, not many studies of this kind have been conducted. The lack of previous studies of this kind in the South African context poses as a limitation for this study.

## Conclusion

This study indicated that basic HIV transmission knowledge was good in this population of mental health care users; however, the average score remained lower than those found in the general population. This confirmed that there is a need for psychiatric patients to receive ongoing psychoeducation about HIV transmission and prevention. This study also showed that patient personal factors such as age, level of education, employment status and marital status are associated with higher basic HIV transmission knowledge scores.

Whilst this study has revealed no significant statistical difference in HIV knowledge with gender and race, literature has indicated inconsistencies in research, with factors such as poverty and development identified as contributing factors to the level of HIV knowledge and infection.

The South African HIV Clinicians Society’s guidelines recommend that all patients with mental disorders, be it in- and/or out-patients, voluntary or involuntary patients admitted under the *Mental Health Care Act*, be offered a HIV test coupled with HIV prevention and risk-mitigating education including access to condoms.^37^ Furthermore, the research literature on HIV prevention interventions shows that intensive, small-group interventions that focus on risk-mitigating areas such as HIV knowledge, attitudes, and motivations, behavioural and cognitive skills can produce short-term reductions risky sexual behaviour among psychiatric patients. It is evident that because of all the impairments faced by mentally ill patients, interventions must allow for long-term retaining of information, or ongoing psychoeducation with every contact with the health care system.
